# Activation of the IL-4/STAT6 Signaling Pathway Promotes Lung Cancer Progression by Increasing M2 Myeloid Cells

**DOI:** 10.3389/fimmu.2019.02638

**Published:** 2019-11-13

**Authors:** Cuiping Fu, Liyan Jiang, Shengyu Hao, Zilong Liu, Suling Ding, Weiwei Zhang, Xiangdong Yang, Shanqun Li

**Affiliations:** ^1^Department of Pulmonary Medicine, Zhongshan Hospital, Fudan University, Shanghai, China; ^2^Shanghai Institute of Cardiovascular Diseases, Zhongshan Hospital, Fudan University, Shanghai, China

**Keywords:** STAT6, lung cancer, IL-4, macrophage polarization, CD11b

## Abstract

Emerging evidence shows that signal transducer and activator of transcription 6 (STAT6) plays critical roles in tumor development. We previously found high-level expression of STAT6 in human lung adenocarcinoma and squamous cell carcinoma, specifically in infiltrated immune cells located in the lung interstitium. Nevertheless, the role of STAT6 signaling in lung carcinogenesis and lung cancer proliferation and its underlying mechanisms remain unclear. This study aimed to investigate the role of STAT6 and the interaction between STAT6 and the tumor microenvironment in pulmonary tumorigenesis. We established a murine model of primary lung carcinogenesis in STAT6-deficient (STAT6^−/−^) and STAT6 wild-type (WT) BALB/c mice using the carcinogen urethane. Two-month-old male mice were intraperitoneally injected with urethane (1 g/kg) dissolved in phosphate buffered saline (PBS). Primary tumors were monitored *in vivo* by positron emission tomography scanning. At 4, 6, and 9 months after urethane injection, lung tumors were harvested from the STAT6^−/−^ and WT mice for analysis. Small interfering RNA was used to downregulate the expression of *STAT6* in tumor cells. Fluorescence activated cell sorting analysis was used to analyze fluorescence-conjugated cell markers. Transwell assays were used in coculturing experiments. STAT6 protein expression was detected by Western blotting, immunohistochemistry, and immunofluorescence. STAT6 mRNA expression was detected by quantitative real time-polymerase chain reaction. Cell Counting Kit-8 and colony formation assays were performed to evaluate cell proliferation. We detected high expression of STAT6 in CD11b^+^ cells of lung carcinoma. Our results indicate that STAT6 deficiency inhibits carcinogen-induced tumor growth and improves prognosis. STAT6 deficiency also decreased the mobilization and differentiation of CD11b^+^ cells. STAT6 deficiency in CD11b^+^ cells but not tumor cells decreased interleukin (IL)-4 secretion and the differentiation of CD11b^+^ cells into M2 macrophage cells. In conclusion, our findings indicate that IL-4/STAT6 signaling in CD11b^+^ cells promotes lung cancer progression by triggering an IL-4 positive feedback loop and increasing M2 myeloid cells. STAT6 may be a new therapeutic target for the prevention and treatment of lung cancer.

## Introduction

Lung cancer is one of the most common malignant tumors worldwide with a relatively poor 5-year survival rate of ~15% (1, 2). Although new therapeutic approaches have been applied to lung cancer and the number of people who smoke has decreased, the overall survival rate of lung cancer has not improved significantly (3). It is anticipated that lung cancer mortality would continue to increase unless more efficacious therapies become available. The key problem is that the mechanism underlying lung carcinogenesis has not been elucidated.

Carcinogenesis is tightly associated with the tumor microenvironment (TME). The tumor and its surrounding microenvironment are closely interrelated; tumors may influence their microenvironment by releasing extracellular signals that promote tumor angiogenesis and induce a peripheral immune tolerance, while immune cells in the microenvironment could affect the growth and evolution of cancerous cells through immuno-editing (4). Transcriptional activation by the signal transducer and activator of transcription (STAT) family of proteins is primarily activated by membrane receptor-associated Janus kinases. Dysregulation of the STAT signaling pathway is frequently observed in primary tumors and leads to increased angiogenesis, enhanced survival of tumors, and immunosuppression (5). Studies have shown that STAT family proteins are involved in the development and function of the immune system and play a critical role in maintaining immune tolerance and tumor surveillance (6–9). Recently, a member of the STAT family, STAT6, has received considerable attention in the area of tumor growth and metastasis. Significantly higher STAT6 immunoexpression level was observed in all histopathological non-small-cell lung cancer (NSCLC) subtypes (squamous cell carcinoma, adenocarcinoma, and large-cell carcinoma), especially higher expression found in squamous cell carcinoma than in large-cell carcinoma (10, 11).

The STAT6 signaling pathway is highly activated in tumors and has been shown to promote tumor metastasis in colorectal cancer and melanoma carcinoma (12, 13). In mammary cancer, STAT6 knockout (KO) mice are 10 times more resistant to lung cancer metastasis than wild-type (WT) mice (14). STAT6 is also activated in invasive T-cell lymphoma, primary mediastinal large B lymphoma, and pancreatic cancer (15). STAT6 is highly expressed in 54–55% of NSCLC, and the negative regulator of STAT6-suppressor of cytokine signaling-3 is reduced in squamous cell lung carcinoma (9). These observations imply a tumor-promoting role for STAT6 in lung cancer. Activation of the STAT6 signaling pathway is also important for macrophage function and required for alternative (M1 and M2) activation of macrophages, which affects inflammation and tumor growth (16). Interleukin (IL)-4 and IL-13 are known to activate the STAT6 signaling pathway by promoting transcription of STAT6-responsive genes (17, 19). However, the role of IL-4 in tumor growth under the condition of STAT6 inactivation is unclear.

The exact role of STAT6 in lung cancer carcinogenesis and progression has not been verified. Our previous studies showed that STAT6 expression is higher in lung carcinoma than para-carcinoma tissues (unpublished data). In the present study, we established a urethane-induced primary lung cancer model in STAT6 knockout mice to explore the effects of STAT6 on lung cancer and to further investigate the interaction between STAT6 and the pulmonary TME. With the high mortality of lung cancer, elucidating the exact mechanism underlying lung cancer progression will contribute to the development of better preventive and treatment strategies for this disease.

## Materials and Methods

### Human Tissue Samples

This study was approved by the institutional review board of Zhongshan Hospital, and written informed consent was obtained from all patients. Lung tumor tissues were obtained from Shanghai Zhongshan Hospital affiliated to Fudan University in 2015 from 36 patients with lung adenocarcinoma. The cohort comprised 16 squamous, 16 adenocarcinoma, 2 mucinous carcinoma, and 2 large-cell lung cancer patients. All lung cancer samples used were NSCLC, and no chemotherapy or radiotherapy was given to patients before surgery. Lung cancer tissue obtained from these 36 patients were made into tissue microarray, 10 sections from each sample were chosen randomly, and 10 images per slide were examined to determine the quick score (QS). All pathology samples were examined independently by two pathologists from Zhongshan Hospital affiliated to Fudan University. Peripheral blood mononuclear cells (PBMC) were obtained from five health donors.

### Animals and Primary Lung Tumor Models

Mice were maintained and bred in the animal facility of Fudan University according to the National Institutes of Health Guidelines for the Humane Treatment of Laboratory Animals. All animal procedures were approved by Fudan University Institutional Animal Care and Use Committee in accordance with the Helsinki Declaration of 1975. WT BALB/c and STAT6 knockout (STAT6^−/−^) BALB/c mice were obtained from the Nanjing Model Animal Center (4, 18). All experiments were conducted in 2-month-old male mice in pathogen-free facilities. Male STAT6^−/−^ mice and their WT littermates were treated with urethane (1 g/kg, Sigma, St. Louis, MO, USA) in total 0.2 ml by intraperitoneal injection (19). The mice in the vehicle groups were injected with the same volume of PBS instead of urethane. Lung tumors were harvested from the mice at 4, 6, and 9 months after urethane injection. No animals or tumors were excluded from the analysis. No formal randomization was performed, and all analyses were performed against the entire data set in an unbiased manner.

### Cells

CD11b^+^ cells were isolated from WT and STAT6^−/−^ mice using magnetic beads. Bone marrow cells and spleen cells from WT and STAT6^−/−^mice were collected and made into single-cell suspensions as previously described (20, 21). Briefly, under aseptic condition, femur from mice was removed from the perifemoral tissue, and the bone marrow cavity was kept closed and intact. One end of the femoral head was cut off, and a disposable sterile syringe was inserted into the cavity, and complete DMEM medium was used to rinse the bone until the bone marrow cavity turned white. The collected liquid was centrifuged at 1,200 rpm, and then the supernatant was removed. After lysis of red blood cells (RBC) by 1 × lysis buffer, resuspension of bone marrow cells was performed using PBS. Bone marrow-derived macrophages from WT and STAT6^−/−^mice were isolated as previously described (22). A549, H1299, normal human bronchial epithelial (HBE), human pulmonary microvascular endothelial cell (HPMEC), human embryonic lung fibroblast (HELF), BEAS2, LLC1, SPC-A1, and SK-MES-1 cell lines were obtained from the Chinese Academy of Sciences, Shanghai Institute of Biochemistry and Cell Biology (Shanghai, China). HELF, BEAS2, LLC1, SPC-A1, and SK-MES-1 cells were cultured in DMEM (Gibco, Waltham, MA, USA) supplemented with 10% fetal bovine serum (FBS) and 1% penicillin–streptomycin, and maintained in DMEM supplemented with L-glutamine (2 mmol/L), HEPES (10 mmol/L), 2-mercaptoethanol (20 mmol/L), streptomycin (150 U/ml), penicillin (200 U/ml), and 10% heat-inactivated FBS (all from Gibco). HPMECs were cultured in endothelial cell medium with endothelial cell growth supplement (Sciencell, Carlsbad, CA, USA). Cells were maintained at 37°C and 5% CO_2_ in a humid environment.

### Micro-PET Scanning of *in vivo* ^18^F-FDG

The mice were fasted for at least 12 h prior to inoculation with ^18^F-fludeoxyglucose (^18^F-FDG). The mice were anesthetized with inhaled isoflurane (3% induction and 1–1.5% maintenance) to eliminate physical movements for positron emission tomography (PET) scanning (Metis, Shandong Maideyinghua Animal Company, China). The parameters used were field of view (FOV) (transaxial): 80 mm; FOV (axial): 60 mm; spatial resolution: <1.3 mm; content rate of scattered radiation: 6.3%; peak value: 226 Kcps; sensitivity 4.3% (380–640 Kev).

### Knockdown of STAT6 in Tumor Cells

Single-stranded RNA-oligonucleotides were synthesized and 20-OH deprotected at Genepharma, Inc. (Shanghai, China). The design of the siRNAs followed the guidelines according to Tuschl (23). The numbering of the siRNAs correlates with the position of the siRNA sequence within the EMBL U16031.1 STAT6 sequence version. The sense sequence of the siRNAs was 5′- CCAAGACAACAAUGCCAAATTUUUGGCAUUGUCUUGGTT-3′(si502). Control experiments were performed with siRNA, and the sense strand (5′-FITCUUCUCCGAACGUGUCACGUTTdTdT-3′) was labeled with 5′-fluorescein isothiocyanate (FITC). This control was added to evaluate transfection efficiencies. We confirmed that this siRNA could efficiently reduce the expression of STAT6 (Supplementary Figure 1). For the transfection experiments, 4 × 10^5^ cells per well were seeded in a six-well plate and incubated overnight in complete growth medium to reach 50–70% confluency. Cells were transfected with 100 nM siRNA in the presence of 100 μl reagent (Santa Cruz Biotechnology, Santa Cruz, CA, USA) in 1 ml of transfection medium. The transfection efficiencies were determined 12 h after the addition of control siRNA by fluorescence microscopy. Cell proliferation and cell cycle analyses were performed by the Cell Counting Kit-8 assay (CCK8) and fluorescence-activated cell sorting (FACS), respectively, 12 h after transfection.

### Cancer Transplantation in Nude Mice

Four-week-old nude mice were purchased from Slac Laboratory Animal Company (Shanghai, China) and housed individually in a light-controlled room (light: 7:00–19:00 h, dark: 19:00–7:00 h) with a temperature of 24 ± 1°C and a relative humidity of 55% ± 5 at the Laboratory Animal Center of Fudan University. The mice were provided with an *ad libitum* supply of filtered pathogen-free air, food, and water. LLC1 cells in logarithmic phase were made into a single-cell suspension of 1 × 10^6^/100 μl. CD11b^+^ cells from BALB/c and STAT6^−/−^ mice were sorted into a single-cell suspension of 1 × 10^6^/100 μl. Cell suspensions (100 μl LLC1 cells plus 100 μl CD11b^+^ cells, or only 100 μl LLC1 cells for the control group) were injected into the left subcutaneous anterior axillary of nude mice under aseptic conditions. After 10 weeks, the mice were euthanized, and the tumors were removed and weighed.

### Antibodies and Immunofluorescence Staining

The following antibodies were used in the current study: STAT6 antibody, CD11b antibody, CD68 antibody, and CD163 antibody (all from Abcam, Cambridge, MA, USA). Tissue samples from STAT6^−/−^ or WT mice were fixed with 4% paraformaldehyde for 12 h followed by 30% sucrose overnight. Texas Red-conjugated rabbit-specific secondary antibody and/or FITC-conjugated mouse-specific secondary antibody (Biolegend, London, UK) were used for immunofluorescence analysis. CD68 and CD163 fluorescence was observed, and images were captured with a Leica TCS SP8 system (Leica, Allendale, NJ, USA).

### Cell Coculture Experiments

Transwell assays were used in the coculture experiments. For the four types of coculture experiments, the cells on the upper and lower side of the filter, respectively, were tumor cells and spleen cells, tumor cells and bone marrow cells, CD11b^+^ cells and tumor cells, and tumor cells and CD11b^+^ cells. Assays were performed as described previously (24). Briefly, using the tumor and spleen cell coculture as an example, a 24-mm Transwell with a 0.4-μm pore polyester membrane insert was used, 1 × 10^5^ of spleen cells from either WT or STAT6^−/−^ mice were placed in the lower side of the filter, and the same number of tumor cells was placed in the upper side. After 48 h of coculture with the tumor cells, the spleen cells were collected for FACS analysis. For tumor proliferation experiments, 1 × 10^5^ of CD11b^+^ cells from WT and STAT6^−/−^ mice were placed in the lower side of the filter, and the same number of tumor cells from the control group was placed in the upper side of the filter. Tumor cells in the lower side of the filer (1 × 10^6^) were used for the CCK8 assay after 12, 24, 48, and 72 h of coculture. For the colony formation assay, 50 tumor cells in the lower side of the filter were stained with Giemsa after a 7-day coculture. For all experiments, transmigrated tumor cells on the lower side of the filter were imaged (Olympus IX81, Oerzen, Germany) and quantified with ImageJ software. Each experiment was performed at least three times with a minimum of six wells per condition.

### Antibodies and FACS Analysis

PE-conjugated lineage-specific antibodies (CD3, CD25, F4/80), APC-conjugated CD206 antibody, PercPcy5.5-conjugated Ly6C and CD4 antibodies, and FITC-conjugated CD8 and CD11b antibodies were purchased from BD Pharmingen (San Jose, CA, USA). For FACS analysis, the isolated cells were washed with PBS and then incubated with the anti-mouse antibodies described above for 40 min at 4°C to achieve specific binding. Single-cell suspensions were evaluated by multi-color flow cytometry using an LSRII flow cytometer (BD Biosciences, San Jose, CA, USA). Data were analyzed using the FlowJo 10 software (BD Biosciences).

### Quantitative Real Time-Polymerase Chain Reaction (RT-PCR)

For quantitative RT-PCR analysis, total RNA was extracted from the tissues of STAT6^−/−^ and control mice using Trizol (Invitrogen, Waltham, MA, USA). High-fidelity cDNA was generated from each RNA sample with Superscript III cDNA amplification System (Invitrogen). Quantitative RT-PCR reaction samples were prepared as a mixture with the Quantitect SYBR Green PCR kit (Qiagen, Dusseldorf, Germany) following the manufacturer's instructions. Reactions were performed using a Prism 9700 PCR machine (Applied Biosystems, Waltham, MA, USA). The PCR conditions were as follows: 95°C for 30 s followed by 45 cycles of 95°C for 15 s, 55°C for 30 s, and 72°C for 30 s. The primer sequences used are shown below (Table 1).

**Table 1:** Primer sequences of genes used in this study.

**Gene**	**Forward**	**Reverse**
GAPDH	5′ -CCACTCACGGCAAATTCAAC-3′	5′ -GGAGAAGGCGTTTGCTTAGTT−3′;
iNOS	5′ -CTCTACAACATCCTGGAGCAAGTG-3′	5′ -ACTATGGAGCACAGCCACATTGA-3′
Arginase1	5′ -GAACCCAACTCTTGGGAAGAC-3′	5′ -GGAGAAGGCGTTTGCTTAGTT−3′
IL-10	5′ -GCCTTATCGGAAATGATCCA-3′	5′ -GAACCCAACTCTTGGGAAGAC-3′
IL-6	5′ -CCGGAGAGGAGACTTCACAG-3′	5′ -CAGAATTGCCATTGCACAAC-3′
IL-1β	5′ - CTGCTTCCAAACCTTTGACC-3′	5′ -AGCTTCTCCACAGCCACAAT-3′
STAT6	5′ -TTCTGCCAAAGACCTGTCCAT-3′	5′ -CTGTCCTCTACCATAGTCACA-3′

*IL, interleukin; STAT6, signal transducer and activator of transcription 6*.

### Hematoxylin-Eosin (H&E) Staining, Immunofluorescence (IF), and Immunohistochemical (IHC) Analyses

Formalin-fixed paraffin-embedded (FFPE) tissue sections were dewaxed, hydrated, and heated for 10 min in a conventional pressure cooler, treated with 3% hydrogen peroxide for 20 min, and incubated with normal goat serum for 30 min. Subsequently, the sections were incubated with antibodies overnight (1:100 anti-STAT6, 1:200 anti-CD11b). After washing, the sections were incubated with biotin-labeled secondary antibody at 37°C for 60 min. The color reaction was developed with 3,3′ -diaminobenzidine tetrahydrochloride. Slides were counterstained with hematoxylin and then coverslips were applied. The IHC was independently assessed by two pathologists who were blinded to the STAT6 genotype and clinicopathological data. The multiplicative QS method that measures both the intensity and extent of cell staining was applied to assess STAT6 expression. In brief, the proportion of positive cells was estimated and given a percentage score on a scale from 1 to 6 (1 = 1–4%; 2 = 5–19%; 3 = 20–39%; 4 = 40–59%; 5 = 60–79%; and 6 = 80–100%). The average intensity of the positively stained cells was given an intensity score from 0 to 3 (0 = no staining; 1 = weak staining, 2 = intermediate staining, and 3 = strong staining). The QS was then calculated by multiplying the percentage score by the intensity score to yield a minimum value of 0 and a maximum value of 18. Based on the QS, STAT6 expression was graded as low (score 0–9) or high (score 10–18). CD11b expression was scored by the same QS method. HE staining was conducted as previously described (25).

### *In vitro* Cell Proliferation Assay

Tumor cells (4 × 10^3^) of si-control and si-STAT6 were seeded on a 96-well tissue culture-treated plate (Corning, New York, NY, USA) in 100 μl complete medium containing 2 μg/ml puromycin to maintain knockdown. Cells were incubated at 37°C in a humidified incubator with 5% CO_2_. CCK8 solution (10 μl, Yisheng, Shanghai, China) was then added to different wells with cells grown for 24, 48, and 72 h, and the plate was incubated at 37°C for 1 h. Absorbance at 490 nm was then measured to quantify the amount of viable cells in each well. The experiments were performed in triplicates.

### Cytokine Assay

CD11b^+^ cells at 5 × 10^5^ cells/well/2 ml growth medium (DMEM, 10% Fetal Clone I, 1% penicillin, 1% streptomycin, and 1% Glutamax) (Gibco, Thermo Fisher Scientific, Waltham, MA, USA) were cocultured with LLC1 tumor cells or the same number of CD11b^+^ cells. Culture medium was collected after 48 h. Blood serum from mice and bronchoalveolar lavage fluid (BALF) were assayed in triplicate using an ELISA kit from R&D Systems (Oakville, Canada) according to the manufacturer's guidelines. BALF was obtained as previously reported (26).

### Statistical Analyses

Data from at least three independent experiments and five mice per group are presented as mean ± SD. Statistical differences between groups were evaluated by the Student's *t*-test. Normality distribution was addressed before using unpaired Student's *t*-tests, one-way ANOVA, or Kruskal-Wallis, as appropriate according to the distribution of the data. *P* < 0.05 was considered statistically significant.

## Results

### STAT6 Is Highly Expressed in Interstitial Cells and Immune Cells of Lung Carcinoma

**Figure 1:**
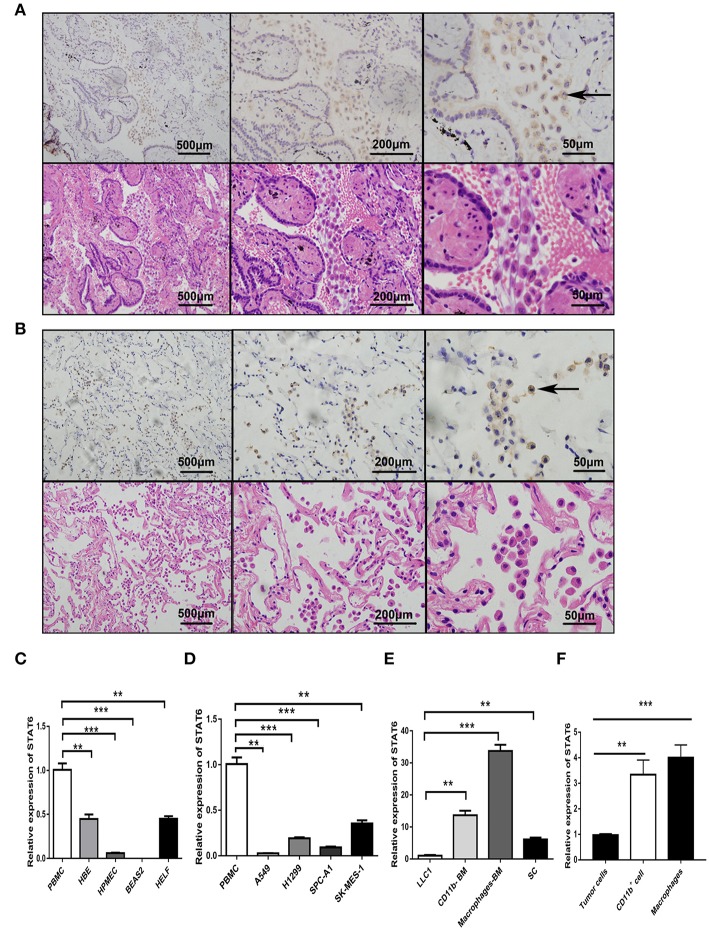
Expression of signal transducer and activator of transcription 6 (STAT6) in lung cancer cells. **(A)** Immunohistochemical (IHC) staining of STAT6 in lung squamous carcinoma and corresponding hematoxylin and eosin (H&E)-stained images. **(B)** IHC staining of STAT6 in lung adenoma carcinoma and corresponding H&E-stained images. **(C)** Comparison of STAT6 mRNA expression between peripheral blood mononuclear cells (PBMCs) and human-derived lung cells. Human-derived lung cell lines bought from ATCC, including normal human bronchial epithelial (HBE), human pulmonary microvascular endothelial cell (HPMEC), human embryonic lung fibroblast (HELF), and human bronchial cell line BEAS2. **(D)** Comparison of STAT6 mRNA expression between PBMC cells and lung cancer-derived cell lines. **(E)** Comparison of STAT6 mRNA expression between immune cells and tumor cells. Spleen cells (SC) were obtained from 6-week old BALB/c mice and removed red blood cells. CD11b^+^ cells and macrophage cells were obtained from bone marrow-derived cells of the same mice after removing red blood cells. **(F)** Comparison of STAT6 mRNA expression between cells. Tumor cells separated from primary tumor tissue induced by urethane, and CD11b^+^ cells and macrophages from the bone marrow of WT mice after urethane inoculation (at time point of 6 months). Data are presented as mean ± SD of one representative experiment. Similar results were seen in three independent experiments. ^**^P < 0.01, ^***^P < 0.001.

**Figure 2:**
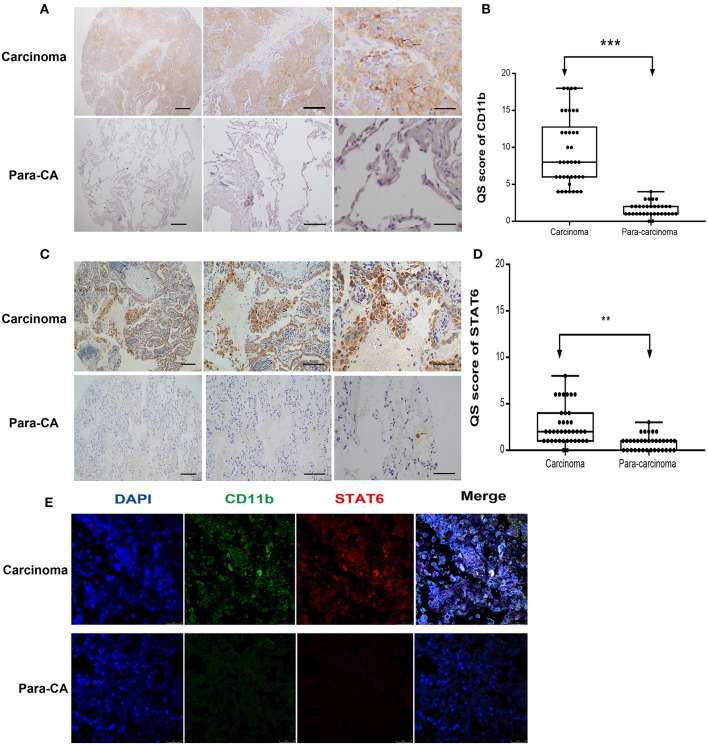
Expression of signal transducer and activator of transcription 6 (STAT6) in CD11b^+^ cells in interstitial cells of carcinoma tissue. **(A)** Immunohistochemical (IHC) staining of CD11b in para-carcinoma and carcinoma tissues of lung cancer patients. Bars represent 2 mm, 500 μm, and 200 μm, respectively. The same magnification is shown in the same column of images. Representative figure of pulmonary adenocarcinoma in the lung cancer patients was shown in Supplementary Figure 5. **(B)** IHC score of CD11b in tissue microarrays of carcinoma and para-carcinoma in patients with lung cancer. **(C)** IHC staining of STAT6 in para-carcinoma and carcinoma tissues of lung cancer patients. Bars represent 2 mm, 500 μm, and 200 μm, respectively. The same magnification is shown in the same column of images. **(D)** IHC score of STAT6 in tissue microarrays of carcinoma and para-carcinoma in 36 patients with lung cancer. **(E)** Confocal scanning of STAT6 and CD11b expression in human lung tissue. Unpaired Student's *t*-tests were used unless noted otherwise. ^**^P < 0.01, ^***^P < 0.001.

To assess the role of the STAT6 signaling pathway in human lung carcinogenesis, STAT6 expression in a cohort of 36 patients with NSCLC was examined by immunological lung tissue microarray analysis. STAT6 protein expression is significantly increased in lung carcinoma tissue compared to para-carcinoma tissue. Within the lung adenocarcinoma and squamous carcinoma tissues, STAT6 expression is higher in interstitial cells than that in epithelial cells (Figures 1A,B). Quantitative RT-PCR revealed that STAT6 mRNA expression is higher in PBMCs than in the normal human lung cell lines HBE, HPMEC, BEAS-2B, and HELF (Figure 1C). STAT6 mRNA expression is also higher in PBMCs than in the human lung cancer cell lines A549, H1299, SPC-A1, and SK-MES-1 (Figure 1D). These results indicate that STAT6 expression is higher in immune cells such as PBMCs, but not in parenchymal cells such as fibroblasts or epithelial cells. In 6-week-old WT BALB/c mice, STAT6 mRNA is highly expressed in immune cells, specifically CD11b^+^ and CD11b^+^ F4/80^+^ bone marrow-derived macrophages (Figures 1E,F). Immunological microarray analyses revealed that in lung carcinoma tissue, CD11b^+^ cells are increased and the IHC scores of CD11b and STAT6 are higher than in lung para-carcinoma tissue (Figures 2A–D). Laser confocal imaging of human lung cancer sections showed that STAT6 is mainly expressed in the interstitial CD11b^+^ immune cells (Figure 2E). The different expression levels of STAT6 in carcinoma and para-carcinoma tissues suggest that STAT6 might play an important role in lung cancer. Furthermore, data from the UCSC Cancer Genomics Browser^1^ indicate that STAT6 is highly expressed in many types of carcinoma including thyroid, breast, and lung cancer. A higher level of STAT6 expression is correlated with poorer survival (Supplementary Figure 2).

### STAT6 Deficiency Inhibits Carcinogen-Induced Tumor Growth and Improves Prognosis

**Figure 3:**
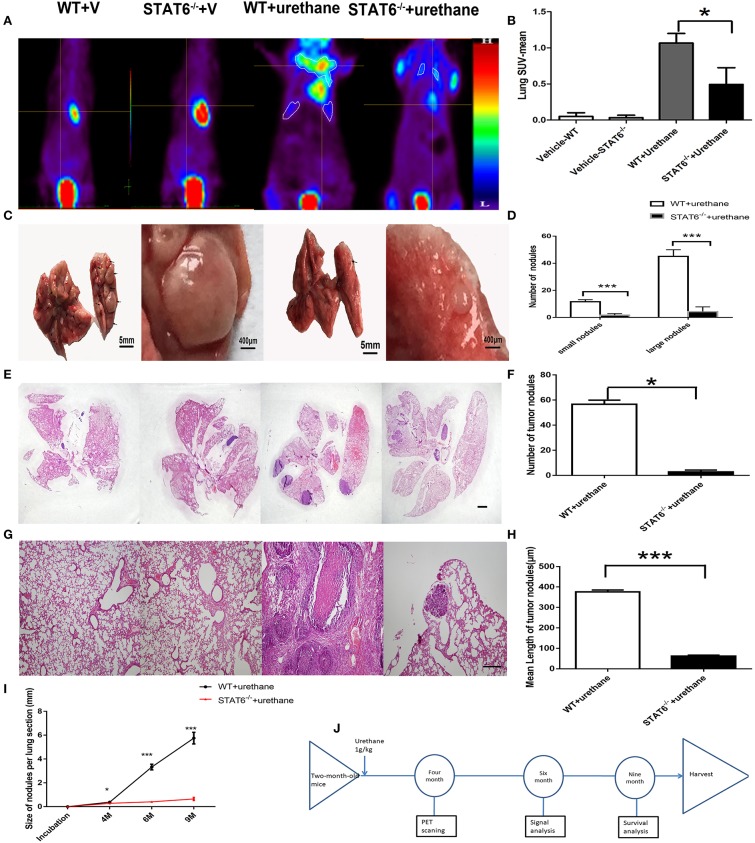
Effect of signal transducer and activator of transcription 6 (STAT6) deficiency on primary tumor growth. **(A–H)** Show results at 6 months after urethane inoculation. **(A,B)** Representative images and quantitative analysis of lung nodules of STAT6^−/−^ mice or WT littermates detected by PET/MR at the time point of 4 months after inoculation of urethane. Urethane was intraperitoneally injected at 1 g/kg. **(C)** Facade of the lung from two groups; the two images on the left show WT tumor-bearing mice and the images on the right show STAT6^−/−^ tumor-bearing mice. **(D)** Number of small (<300 μm) and large nodules (more than 300 μm). **(E)** Hematoxylin and eosin (H&E) staining of overall lung sections. The bar represents 5 mm. The four images have the same magnification. From left to right: WT+V, STAT6^−/−^+V, WT+urethane, STAT6^−/−^+urethane. **(F)** Quantification of lung nodules. **(G)** H&E-stained lung sections. The bar represents 2 mm. The four images have the same magnification. From left to right: WT+V, STAT6^−/−^ +V, WT+urethane, STAT6^−/−^+urethane. **(H)** Quantification of mean length in the lung section. **(I)** Size of tumor nodules at 4, 6, and 9 months. WT+V: WT mice with PBS (vehicle) inoculation; STAT6^−/−^+V: STAT6 knockout mice with PBS (vehicle) inoculation. WT+urethane: WT mice with urethane inoculation; STAT6^−/−^+urethane: STAT6 knockout mice with urethane inoculation. **(J)** The timeline of this experiment. Briefly, 2-month-old mice were induced with primary carcinogen by urethane (1 g/kg), then were harvested for biochemistry analysis 6 months later of urethane, and part of mice were kept to 9 months for survival analysis. All mice were harvested at 9 months later of urethane. Data are presented as mean ± SD of one representative experiment. Similar results were obtained in three independent experiments. Unpaired Student's *t*-tests were used unless noted otherwise. ^*^P < 0.05, ^***^P < 0.001.

To explore the specific role of STAT6 in lung carcinogenesis, a primary lung tumor model was established in WT and STAT6^−/−^ mice by induction with a single high dose of urethane injection. Urethane is a good kind of carcinogen in mice. Tumors were established in 94.44% of WT mice and 50% of STAT6^−/−^ mice. The mean-SUV value by PET showed less F^18^ accumulation in the lung of STAT6^−/−^ mice compared to WT mice (Figures 3A,B), and relatively decreased lung tumor load was shown. We observed that a primary tumor could be detected by PET 4 months after urethane injection. Significant reductions in the number and size of tumors were observed in primary tumor-bearing STAT6^−/−^ mice compared to WT littermates (Figure 3C). Nodules smaller and larger than 300 μm were designated as small and large nodules, respectively. The results showed that the nodules in the STAT6^−/−^ mice are fewer and smaller (Figure 3D) than the nodules in their WT littermates. HE staining of whole-lung sections further confirmed the reduced tumor sizes and reduced inflammation response around tumor nodules in STAT6^−/−^ tumor-bearing mice (Figures 3E–H). During the 9-month observation, the suppression of tumor progression in the STAT6^−/−^ mice became increasingly obvious (Figure 3I), and the survival rate is higher in the STAT6^−/−^ mice than in WT mice, 94.44 vs. 83.33%, respectively. The timeline of this experiment was shown in Figure 3J. The rate of positive TUNEL staining is higher and the rate of positive Ki67 staining is lower in the tumors from STAT6^−/−^ mice than WT mice (Supplementary Figure 3), which suggest less proliferation and increased apoptosis in tumors from STAT6^−/−^ mice. These results indicate that STAT6 deficiency suppresses tumor progression.

### Decreased Mobilization and Differentiation of CD11b^+^ Cells in STAT6^−/−^ Tumor-Bearing Mice

**Figure 4:**
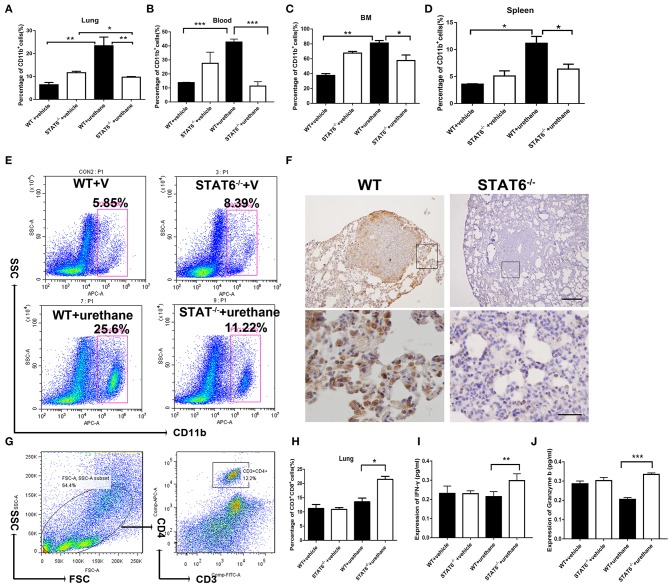
CD11b^+^ cell staining and analysis in tumor-bearing mice. **(A–D)** Quantification of the percentages of CD11b^+^ cells in the blood, lung, bone marrow, and spleen in WT+vehicle, signal transducer and activator of transcription 6 (STAT6) ^−/−^+vehicle, WT+urethane, and STAT6^−/−^+urethane groups. **(E)** Representative flow cytometry analysis results of CD11b^+^ cells in the lung of STAT6^−/−^ and WT mice 6 months after vehicle or urethane inoculation. **(F)** Immunohistochemical (IHC) staining of CD11b^+^ in the lung. The upper and lower bars represent 500 and 200 μm, respectively. Images in the same row are of the same magnification. **(G)** Flow cytometry analysis diagram of CD3-CD4 in mice. **(H)** Percentage of CD3^+^CD8^+^ cells by flow cytometry analysis. **(I,J)** Expression of cytokines associated with cytotoxic T lymphocytes such as interferon (IFN)-γ and granzyme-b (pg/ml). Data are presented as mean ± SD of one representative experiment. Total number of gated cells was 5 × 10E4. Similar results were obtained in three independent experiments. Unpaired Student's *t*-tests were used unless noted otherwise. ^*^P < 0.05, ^**^P < 0.01, ^***^P < 0.001.

The cellular composition of the lung in STAT6^−/−^ mice and their WT littermates after urethane injection was analyzed. Lung tissue was minced by electronic ultrasonic machine. RBCs were removed using RBC lysis buffer, and then flow cytometry analysis was carried out. Figures 4E,G were done on the whole-lung digest after removing erythrocytes. FACS analysis showed that CD11b^+^ myeloid cells are remarkably expanded in the lung, blood, bone marrow, and spleen of WT mice while reduced in STAT6^−/−^ mice after urethane inoculation (Figures 4A–E). Immunohistochemical staining of the lung of STAT6^−/−^ mice showed reduced CD11b expression around the primary tumor nodules (Figure 4F). CD11b^+^ cells exhibit less inflammatory infiltrate surrounding the primary tumor in STAT6^−/−^ mice compared to WT littermates. The total number of neither CD3^+^CD4^+^ cells nor CD4^+^CD25^+^ T-regulatory cells is altered in this tumor-bearing model (Figure 4G; Supplementary Figure 4). However, the number of CD3^+^CD8^+^ cells is increased, and the levels of cytokines associated with cytotoxic T lymphocytes [interferon (IFN)-γ and granzyme-b] are elevated in STAT6^−/−^ tumor-bearing mice compared to WT mice (Figures 4H–J). The reduced percentage and number of CD11b^+^ cells in the STAT6^−/−^ mice indicate that STAT6 deficiency reduces the recruitment of CD11b^+^ cells to the blood and lung in carcinogen-induced primary lung neoplasm. Therefore, CD11b^+^ cells may be an influencing factor in carcinogen-induced tumor growth in STAT6^−/−^ mice.

### STAT6 Deficiency in CD11b^+^ Cells Suppresses Tumor Proliferation

**Figure 5:**
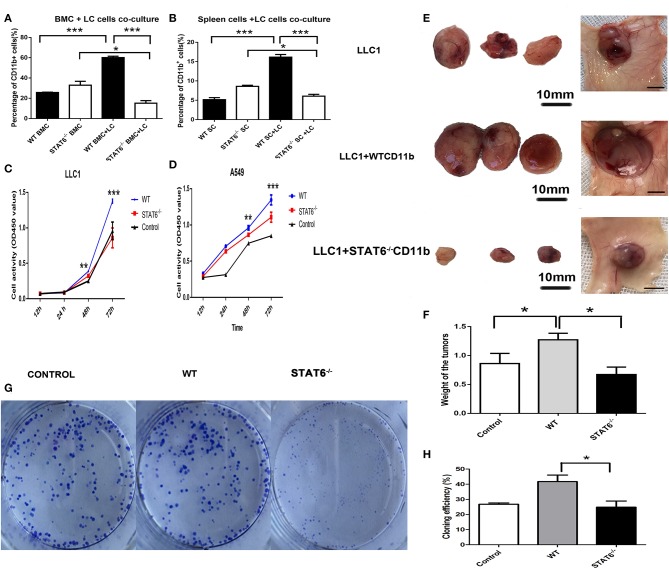
Depletion of signal transducer and activator of transcription 6 (STAT6) in CD11b^+^ cell affects tumor growth. **(A,B)** Lung cancer cells (LLC1) were cocultured with either bone marrow cells or spleen cells, and the percentages of CD11b^+^ cells were analyzed by FACS. **(A)** Percentage of CD11b^+^ cells in bone marrow cells derived from STAT6^−/−^ mice or WT littermates when cocultured with LLC1 cells. **(B)** Percentage of CD11b^+^ cells in spleen cells derived from STAT6^−/−^ mice or WT littermates when cocultured with LLC1 cells. **(C,D)** Proliferation curve of LLC1/A549 cells when cocultured with CD11b^+^ cells derived from mice. WT group indicates coculture of cancer cells with CD11b^+^ cells derived from WT mice; STAT6^−/−^ group indicates coculture of cancer cells with the same number of CD11b^+^ cells derived from STAT6^−/−^ mice as in the WT group; equal numbers of cancer cells were substituted for CD11b+ cells in the control group. **(E)** Facade of tumor volume in the control, WT, and STAT6^−/−^ groups. An equal number of CD11b^+^ cells (1 × 10^6^) derived either from STAT6^−/−^ mice or WT mice was mixed with 1 × 10^6^ LLC1 cells and inoculated subcutaneously into nude mice. An equal number of LLC1 cells (1 × 10^6^) was substituted for the CD11b^+^ cells in the control group. After 10 weeks, tumor sizes and weights were measured. **(F)** Tumor weights in the control, WT, and STAT6^−/−^ groups. **(G,H)** Colony formation of cancer cells (CMT167) cocultured with CD11b^+^ cells derived from either STAT6^−/−^ mice or WT littermates 7 days after the start of the experiment. The control group shows the colony formation of cancer cells cocultured with the same number of cancer cells as CD11b^+^ cells. ^*^P < 0.05, ^**^P < 0.01, ^***^P < 0.001.

To further ascertain the role of CD11b^+^ cells in this primary lung tumor model, LLC1 murine cancer cells were cocultured with either bone marrow cells from WT mice or bone marrow cells from STAT6^−/−^ mice. Interestingly, a marked reduction in the percentage of CD11b^+^ cells was observed in bone marrow cells derived from STAT6^−/−^ mice compared to bone marrow cells from WT in the cocultures with cancer cells (Figure 5A). The cancer cells increase the number of CD11b^+^ cells, while STAT6 deficiency suppresses the formation of CD11b^+^ cells. The same phenomenon was observed in a coculture of cancer cells with spleen cells derived from either STAT6^−/−^ or WT mice (Figure 5B). The results suggest that cancer cells inhibit CD11b^+^ cell proliferation in bone marrow and spleen cells derived from STAT6^−/−^ mice. Furthermore, we explored the effect of CD11b^+^ cells on cancer cell proliferation. Equal numbers of CD11b^+^ cells were isolated from the bone marrow of WT and STAT6^−/−^mice and cocultured with cancer cells (LLC1 and A549) in Transwell plates. Results from a CCK8 assay show less LLC1 cell proliferation when cocultured with STAT6^−/−^ CD11b^+^ cells than with STAT6^+/+^ CD11b^+^ cells (Figure 5C). The same effect was observed in the A549 human lung cancer cells (Figure 5D). We next compared the effects of STAT6^−/−^ CD11b^+^ and STAT6^+/+^ CD11b^+^ cells on tumor growth *in vivo*. Equal numbers of CD11b^+^ cells were isolated from the bone marrow of WT and STAT6^−/−^ mice and mixed with 1 × 10^6^ LLC1 cancer cells and inoculated subcutaneously into nude mice. The control group of nude mice was inoculated with 1 × 10^6^ cancer cells alone. The results show that both the volume (Figure 5E) and weight (Figure 5F) of tumors from STAT6^−/−^ CD11b^+^ cells are less than those from STAT6^+/+^ CD11b^+^ cells, suggesting that CD11b^+^ cells derived from STAT6^−/−^ mice are associated with the reduction of cancer development. To further verify the promotive effect of STAT6 on cancer cell progression, colony formation experiments were carried out in the CMT167 cancer cell line. CMT167 cells show less colony formation when cocultured with STAT6^−/−^ CD11b^+^ cells than when cocultured with STAT6^+/+^ CD11b^+^ cells 7 days after the start of the experiment (Figures 5G,H). Taken together, these results indicate that CD11b^+^ cells from WT mice were related to tumor growth, while CD11b^+^ cells from STAT6^−/−^ mice limited tumor growth.

### STAT6 Promotes M2 and Suppresses M1 Myeloid Cell Polarization in the TME

**Figure 6:**
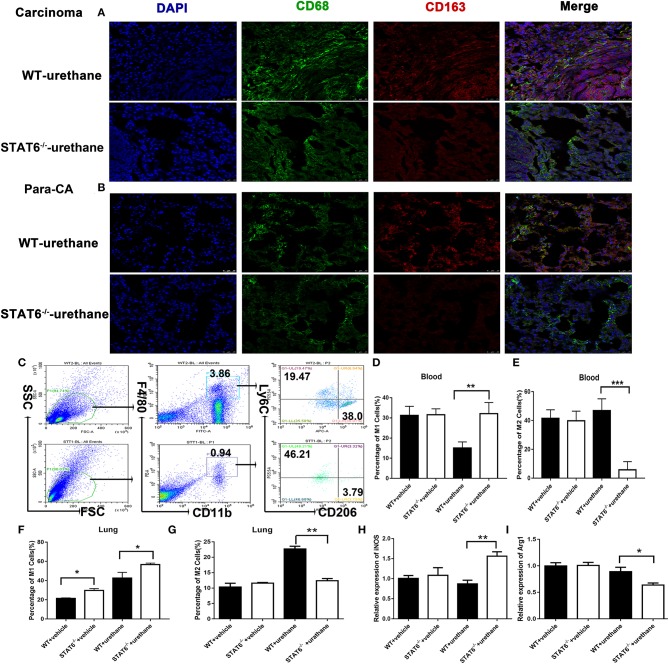
M1 and M2 mobilization in the lung of signal transducer and activator of transcription 6 (STAT6)-deficient mice. **(A,B)** Fluorescent images of CD68 (green) and CD163 (red) in lung carcinoma and para-carcinoma tissues of WT and STAT6^−/−^ tumor-bearing mice. **(C)** Flow cytometry analysis of M1 and M2 cells. **(D–G)**. M1 and M2 cells in WT and STAT6^−/−^ tumor-bearing mice. **(D)** Percentage of M1 cells (CD11b^+^ F4/80^low^ Ly6C^high^CD206^−^) in the blood. **(E)** Percentage of M2 cells in the blood (CD11b^+^ F4/80^high^Ly6C^−^CD206^+^). **(F)** Percentage of M1 cells in the lung. **(G)** Percentage of M2 cells in the lung. **(H)** Relative mRNA expression of iNOS in CD11b^+^cells isolated from the lung. **(I)** Relative mRNA expression of Arg1 in CD11b^+^ cells directly obtained from mice. Total number of gated cells was 10 × 10E4. ^*^P < 0.05, ^**^P < 0.01, ^***^P < 0.001.

We analyzed the Ly6G^+^Ly6C^−^ cells and CD11C^+^ cells among CD11b^+^ cells in the blood, bone marrow, and the lung in WT and STAT6^−/−^ mice at 6 months after urethane induction; however, no significant difference was shown among this kind of cellular population (data shown in Supplementary Figure 6). Our results reveal the possibility of macrophages being the target of STAT6 since STAT6 is highly expressed in macrophages (Figure 1). Previous studies indicate that STAT6 plays a role in macrophage polarization, and a change in this potential could affect carcinogenesis and neoplasm progression (21, 27). We investigated whether changes in macrophage polarization is involved in the enhanced immunity of STAT6^−/−^ mice. Immunofluorescence studies indicated that fewer pan macrophages (CD68^+^CD163^+^) are present in the lung of STAT6^−/−^ tumor-bearing mice than their WT littermates (Figures 6A,B). Flow cytometry analysis showed increased CD11b^+^F4/80^low^Ly6C^high^CD206^−^ M1 cells and decreased CD11b^+^F4/80^high^Ly6C^−^CD206^+^ M2 cells in both the lung and blood of STAT6^−/−^ tumor-bearing mice (Figures 6C–G). CD11b^+^ cells isolated from STAT6^−/−^ mice produce more inducible nitric oxide synthase (iNOS) and less arginase 1 (Arg1) compared to CD11b^+^ cells isolated from WT mice after carcinogen induction when the baseline was the same (Figures 6H,I). Both the decrease in M2 and increase in M1 macrophages contribute to tumor suppression, while M2 macrophages show a greater decrease in CD11b^+^ cells compared to the ascending range of M1 macrophages. These observations indicate that macrophage polarization exists in this primary lung tumor model, and STAT6 might play a key role in macrophage polarization in the TME.

### STAT6 Deficiency in CD11b^+^ Cells but Not Tumor Cells Suppresses IL-4 Secretion

**Figure 7:**
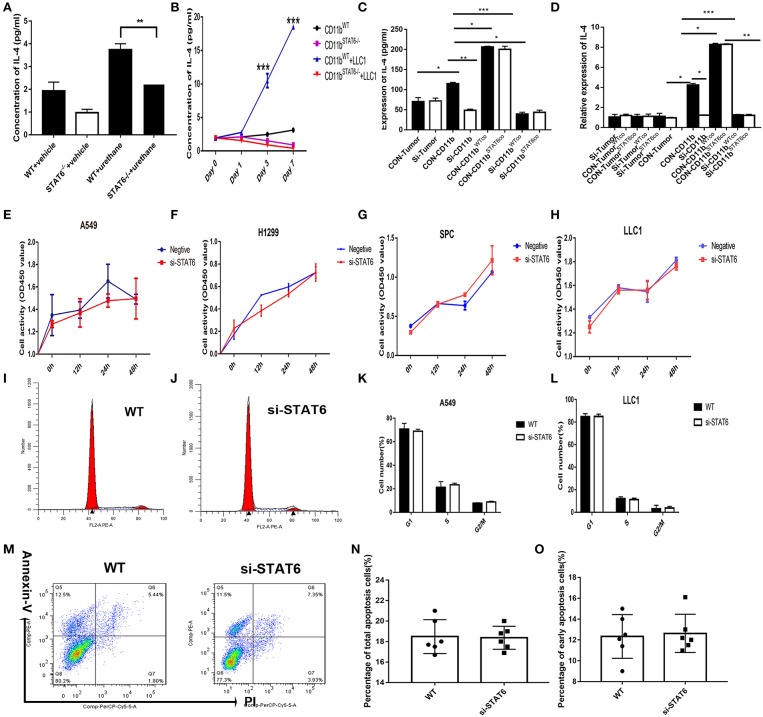
Effect of signal transducer and activator of transcription 6 (STAT6) on IL-4 secretion *in vitro*. **(A)** Concentration of IL-4 (pg/ml) from bronchoalveolar lavage fluid in tumor-bearing wild-type (WT) and STAT6^−/−^ mice. **(B)** Concentration of IL-4 (pg/ml) in the serum when CD11b^+^ cells were cocultured with lung cancer cells (LLC1) using Transwell plates. CD11b^+^ cells were obtained for bone marrow of mice. **(C)** Secretion of IL-4 was measured by ELISA in the cocutured medium of tumor cells [STAT6^+/+^ or STAT6^−/−^ with CD11b^+^ cells (STAT6^+/+^ or STAT6^−/−^)]. CON-Tumor: LLC1 tumor cells. Si-Tumor: STAT6 knockdown in LLC1 cells. CON-CD11b: CD11b^+^ cells. Si-CD11b: CD11b^+^ cells from STAT6^−/−^ mice. CON-CD11b^WTco^: cocultured medium of LLC1 cells with STAT6^+/+^CD11b^+^ cells for analysis of IL-4 expression. CON-CD11b^STAT6co^: cocultured medium of LLC1 cells with STAT6^−/−^CD11b^+^ cells for analysis of IL-4 expression. Si-CD11b^WTco^: cocultured medium of STAT6^−/−^LLC1 cells with STAT6^+/+^CD11b^+^ cells for analysis of IL-4 expression. Si-CD11b^STAT6co^: cocultured medium of STAT6^−/−^LLC1 cells with STAT6^−/−^CD11b^+^ cells for analysis of IL-4 expression. **(D)** mRNA expression of IL-4 was analyzed using RT-PCR. CD11b^+^ cells were isolated from STAT6^−/−^ mice and WT littermates. CON-CD11b: CD11b^+^ cells from bone marrow of WT mice. Si-CD11b: CD11b^+^ cells from STAT6^−/−^ mice. CON-Tumor: LLC1 tumor cells. Si-Tumor: STAT6 knockout in LLC1 cells. CON-Tumor^WTco^: LLC1 cells were separated after 48 h of coculture with CD11b^+^ cells. CON-Tumor^STAT6co^: LLC1 cells were separated after 48 h of coculture with STAT6^−/−^CD11b^+^ cells for analysis of IL-4 expression. Si-Tumor^WTco^: STAT6^−/−^LLC1 cells were separated after 48 h cocultured with STAT6^+/+^CD11b^+^ cells for analysis. Si-Tumor^STAT6co^: STAT6^−/−^LLC1 cells were separated after 48 h of coculture with STAT6^−/−^CD11b^+^ cells for analysis. CON-CD11b^WTco^: STAT6^+/+^CD11b^+^ cells were separated after 48 h of coculture with STAT6^+/+^LLC1 cells. CON-CD11b^STAT6co^: STAT6^+/+^CD11b^+^ cells were separated after 48 h of coculture with STAT6^−/−^LLC1 cells. Si-CD11b^WTco^: STAT6^−/−^CD11b^+^ cells were separated after 48 h of coculture with STAT6^+/+^LLC1 cells. Si-CD11b^STAT6co^: STAT6^−/−^CD11b^+^ cells were separated after 48 h of coculture with STAT6^−/−^LLC1 cells for analysis of IL-4 expression. **(E–H)** CCK8 proliferation analysis of human lung cancer-derived cells (A549, H1299, SPC-A1) and mouse lung cancer-derived cells (LLC1) after genetic knockdown of STAT6. The knockdown efficiencies of si-STAT6 in A549, H1299, SPC-A1, and LLC1 were 85, 90, 97.5, and 88%, respectively, as verified by RT-PCR. **(I,J)** Representative results of cell cycle analysis in WT and si-STAT6 groups. **(K,L)** Comparative percentages of cells in G1, S, and G2/M between WT and STAT6^−/−^ groups in A549 and LLC1 cells. **(M)** Flow cytometry analysis of apoptosis. **(N)** Percentages of apoptotic cells in the WT and STAT6^−/−^ groups (annexin-V-positive, PI-positive or -negative). **(O)** Percentages of early apoptotic cells (annexin-V-positive, PI-negative). si-STAT6, STAT6 knockdown.

We found that IL-4 is significantly reduced in the BALF of STAT6^−/−^ tumor-bearing mice (Figure 7A). Secretion of IL-4 is induced when tumor cells are cocultured with CD11b^+^ cells (Figure 7B). To identify which cells secrete IL-4, the cocultured tumor cells and CD11b^+^ cells were separated to analyze IL-4 expression. IL-4 is consistently reduced in STAT6^−/−^ CD11b^+^ cells compared to WT CD11b^+^ cells when cocultured with tumor cells. We found that reduced IL-4 secretion is mainly attributed to STAT6 deficiency in CD11b^+^ cells but not tumor cells (Figures 7B–D). As STAT6 is mainly expressed in immune cells such as CD11b^+^ cells, we hypothesize that the depletion of STAT6 signaling affects lung tumor cell proliferation through interaction with the TME. As shown in Figure 2, tumor cells express very little STAT6 or do not express STAT6 at all, suggesting that STAT6 biological activity cannot reside predominantly in the tumor cells. To verify this idea, we constructed STAT6 knockdown cell lines *in vitro* via siRNA. We observed that knockdown of the *STAT6* gene in several lung tumor cell lines (A549, H1299, SPC-A-1, and LLC1) resulted in no obvious change in tumor cell activity *in vitro* (Figures 7E–H). Knockdown of STAT6 does not affect cell proliferation (Figures 7I–L) or cell apoptosis (Figures 7M–O). These results indicate that the disruption of the *STAT6* gene alone in tumor cells is insufficient to alter proliferation. Instead, it is STAT6 in CD11b^+^ cells that plays a relatively more important role in tumor cell proliferation.

### STAT6 in CD11b^+^ Cells Promotes Cancer Cell Proliferation by Increasing M2 Myeloid Cells Through Upregulating IL-4

**Figure 8:**
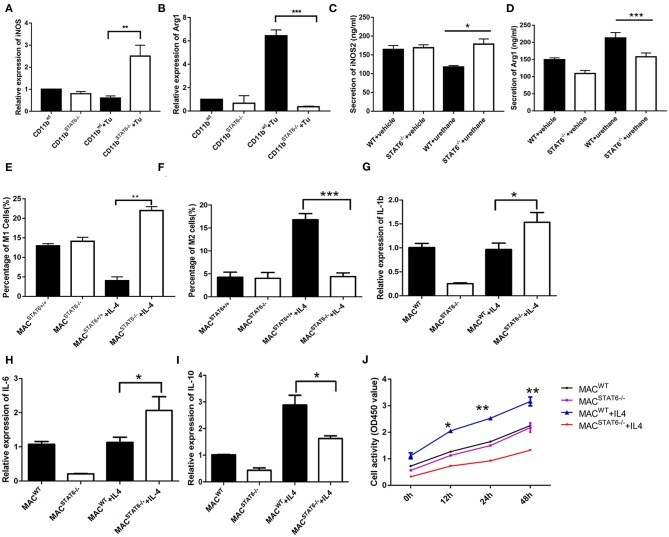
Cytokines play a role in signal transducer and activator of transcription 6 (STAT6) ^−/−^-mediated macrophage polarization. **(A)** Relative mRNA expression of iNOS in CD11b^+^ cells when cocultured with LLC1 cells. **(B)** Relative mRNA expression of Arg1 in CD11b^+^ cells when cocultured with LLC1 cells. **(C,D)** Expression of iNOS and Arg1 in groups of wild type and STAT6^−/−^ mice. **(E)** Percentage of M1 cells in bone marrow-derived macrophages after 7 days of culture with 10 ng/ml exogenous interleukin (IL)-4. **(F)** Percentage of M2 cells in bone marrow-derived macrophages after 7 days of culture with 10 ng/ml exogenous IL-4. **(G)** Relative mRNA expression of IL-1β in bone marrow-derived macrophages after 7 days of culture with 10 ng/ml exogenous IL-4. **(H)** Relative mRNA expression of IL-6 in bone marrow-derived macrophages after 7 days of culture with 10 ng/ml exogenous IL-4. **(I)** Relative mRNA expression of IL-10 in bone marrow-derived macrophages after 7 days of culture with 10 ng/ml exogenous IL-4. **(J)** LLC1 cells (lower plates) were cocultured with macrophages (upper plates) using Transwell plates. Cell proliferation of LLC1 cells was analyzed by CCK8 with or without stimulation by exogenous IL-4. ^*^P < 0.05, ^**^P < 0.01, ^***^P < 0.001.

Since we had established the important role of CD11b^+^ cells in tumor cell proliferation, our next goal was to identify the specific targets of CD11b^+^ cells. To further investigate the mechanism underlying the effect of STAT6 deficiency on the polarization of M1/M2 in the TME, we cocultured LLC1 cancer cells with CD11b^+^ cells derived from the bone marrow of either WT or STAT6^−/−^ mice. A significant reduction of M2 cells and a moderate increase of M1 cells are observed in STAT6^−/−^ CD11b^+^ cells cocultured with LLC1 cells compared to the control (Figures 8A,B) and decreased expression of Arg1 and increased protein expression of iNOS (Figures 8C,D), indicating that STAT6^−/−^ CD11b^+^ cells cause less M2 differentiation and more M1 differentiation in the TME. When IL-4 was added to bone marrow cell-derived macrophages to induce differentiate to M2, a robust increase in Arg1 expression was observed in the control, and this effect was blocked by STAT6 deficiency (Figures 8E,F). Gene expression of M1-associated cytokines IL-1β and IL-6 was increased and of M2-associated IL-10 was decreased (Figures 8G–I). LLC1 cells cocultured with macrophages from STAT6^−/−^ mice exhibit a slower proliferation rate, and exogenous addition of IL-4 further amplifies this difference (Figure 8J). These observations confirm that deficiency of STAT6 decreases M2 polarization and thus suppresses tumor growth. Taken together, our data suggest that STAT6 promotes cancer cell proliferation by altering tumor-macrophage interactions and IL-4 secretion. STAT6 in CD11b^+^ cells increases IL-4 secretion, which in turn increases M2 myeloid cells and promotes cancer cell proliferation.

## Discussion

Westcott et al. (20) reported that Kras is the target-sensitive gene for urethane-induced primary lung cancer. We demonstrated in this study that urethane is a good choice for establishing a lung cancer model in WT and STAT6^−/−^ mice. Here we present data demonstrating that STAT6 is highly expressed in lung carcinoma tissue and predominantly in the interstitial CD11b^+^ cells. In a urethane-induced primary lung cancer model, STAT6 deficiency inhibits tumor growth and improves prognosis. Urethane is an efficient carcinogen of lung cancer (28) that can simulate a human tumor environment comparable with the classic immunosuppressive mammalian model.

Our study focused on the role of bone marrow-derived CD11b^+^ cells in tumorigenesis, and we investigated the interaction of STAT6 with the TME. STAT6 signaling was shown to promote CD11b^+^ cell differentiation into M2 macrophages. Deficiency of STAT6 downregulates CD11b expression in nearby myeloid cells, thereby inhibiting further myeloid differentiation, especially to macrophages, and suppresses the initiation and promotion of cancer cells. We observed decreased CD11b^+^ cells around the primary tumor in the lung and blood. Although STAT6 deficiency has been reported to suppress tumor progression in mammary cancer and colon carcinoma (29), our study reveals a different mechanism of action. Bone marrow-derived macrophages from STAT6^−/−^ mice and their WT littermates present different polarizations, which exert different effects on tumor growth. While both M1 and M2 cells can differentiate from CD11b^+^ cells, STAT6 activation in CD11b^+^ cells promotes differentiation into more M2 and fewer M1 macrophages.

Among myeloid cell types in the lungs, macrophages are the most abundant and perhaps the most pleiotropic (30, 31). CD11b is one of the most important biomarkers of bone marrow-derived myeloid cells, and tumor cells interact with CD11b^+^ cells through various signaling pathways and products secreted in TME (32, 33). Among CD11b^+^ cells, it was reported that myeloid-derived suppressor cells (CD11b^+^Gr1^+^) were increased in lung microenvironment after urethane-induced lung tumor (34, 35). One of the limitations of this study is that while we detected multiple phenotypic changes in tumor-associated macrophages, our strategies were unable to assess direct *in vivo* functions of these cells. Our findings suggest that markedly decreased population of M2 cells could lead to a reduction of CD11b^+^ cells in the lungs of STAT6^−/−^ mice, and increased subpopulation of CD11b^+^ cells in the blood could be an elevated M1 cell population. The TME contributes to increased CD11b^+^ activation and M2/M1 polarization in WT mice, which collectively influence tumor cell proliferation. In contrast, there is decreased CD11b^+^ activation and M2/M1 polarization in STAT6-deficient mice.

Tumor-associated macrophage infiltration is correlated with tumor stage and metastasis (36). Macrophages are important tumor-infiltrating cells and play pivotal roles in tumor growth. Genetic knockdown of STAT6 leads to a reduced expression of Arg1 (37). Depending on micro-environmental cues, these cells could stimulate an inflammatory response by the secretion of pro-inflammatory cytokines or suppress immune responses by releasing high levels of anti-inflammatory cytokines (38). Macrophages participate in immune responses to tumors in a polarized manner; M1 cells promote tumoricidal responses, while M2 cells promote tumor progression. In this study, increased IL-6 and IL-1β expression indicated induction of classic M1 macrophages, whereas decreased IL-10 and IL-4 suggested the downregulation of M2 macrophages (39). It has been reported that M1 macrophages decrease the viability of A549 cells by inducing apoptosis and senescence, while M2 macrophages secrete IL-10 and drive tumor progression (40). The M1 and M2 macrophage subtypes show opposite effects on lung cancer progression. Based on the phenotypic spectrum of these cells, most studies examining the M1/M2 macrophage paradigm in tumors have been performed during the progression stages of established tumors (41). M2/M1 gene expression has been used as a prognostic indicator for lung cancer patients (42). The M2/M1 macrophage density in tumor islets is an independent predictor of survival time in patients with NSCLC (43). Our study showed decreased M2 and increased M1 macrophages in the lungs of STAT6-deficient mice. The reduced differentiation to M2 macrophages in STAT6-deficient mice indicates that macrophage polarization contributes to a suppressed tumor growth state.

Depletion studies in the mammary carcinoma system performed *in vivo* demonstrated that CD8^+^ T cells are essential for tumor rejection by STAT6^−/−^ mice (12). CD8^+^ T cells are increased in STAT6^−/−^ tumor-bearing mice, and decreased M2 and elevated CD8^+^ cytotoxic T lymphocytes exert a synergistic inhibitory effect on tumor proliferation in STAT6^−/−^ mice. Studies have demonstrated that STAT6 deficiency reduces the Th2 response in lung inflammation (44). In melanoma, STAT6 activation increases the Th2 response (45). We did not observe any changes in CD4^+^ cells in the present study. The heightened tumor immunity in STAT6-deficient mice is likely not due to the balance between CD4^+^, Th1, and Th2 cells, which is consistent with the results from other studies (46).

Our study demonstrated for the first time that IL-4 is not only an activator of the STAT6 signaling pathway but also a downstream effector in the TME that acts as a positive feedback factor. IL-4 promotes polarization into the M2-Mϕ phenotype and promotes tumor progression (47). We observed that tumor cells secrete lower levels of IL-4 after being cocultured with STAT6^−/−^ CD11b^+^ cells, indicating that the pathway regulating IL-4 secretion pathway is inhibited by STAT6 deficiency in CD11b^+^ cells but not tumor cells. We confirmed that IL-4 exerts an effect on tumor-associated macrophage polarization. Under the condition of STAT6 deficiency, IL-4 levels are reduced, which results in less polarization toward M2 macrophages. Although STAT6 is one of the most important downstream targets of IL-4, other pathways may function in the absence of STAT6 (48), enabling IL-4 to exert effects on CD11b^+^ cells under STAT6-deficient conditions. A limitation of the present study is that we specifically studied M1 or M2 macrophages differentiated from CD11b^+^ cells. More detailed macrophage subtype data may be necessary for phenotypic analysis in addition to the F4/80 data.

**Figure 9:**
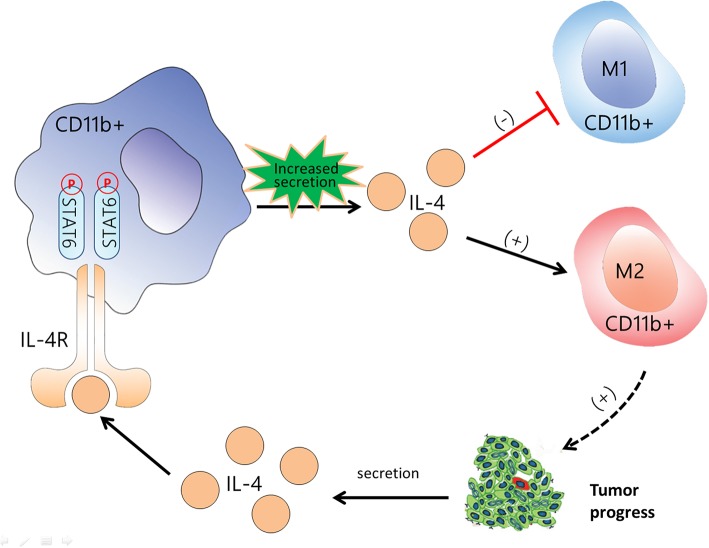
Flow diagram illustrating the main idea of this study. Activation of signal transducer and activator of transcription 6 (STAT6) signaling in CD11b^+^ cells increases the secretion of IL-4, promotes differentiation to M2 myeloid cell, and suppresses M1 polarization. M2 myeloid cells promote cancer cell proliferation and interleukin (IL)-4 secretion in tumor cells, which creates a positive feedback loop. IL-4 binding to the IL-4 receptor begins the next positive feedback loop. The black arrow indicates the promotive effect, and the red line indicates the suppressive effect. CD11b^+^ cells are represented by the red cells among the tumor cells.

In summary, this study showed that activation of STAT6 signaling in CD11b^+^ cells promotes increased IL-4 secretion and M2 myeloid cell polarization. M2 myeloid cells promote cancer cell proliferation and IL-4 secretion in tumor cells, which creates a positive feedback loop, as illustrated in Figure 9. IL-4 binding to the IL-4 receptor begins the next positive feedback loop, which promotes lung cancer progression. Conversely, a deficiency of STAT6 reduces IL-4 secretion and polarization of M2 macrophages, which together contribute to the suppression of lung carcinogenesis. Our findings suggest that STAT6 has the potential as a biomarker in patients with NSCLC, which may contribute to the development of novel preventive and treatment approaches for lung cancer.

## Data Availability Statement

The raw data supporting the conclusions of this manuscript will be made available by the authors, without undue reservation, to any qualified researcher.

## Ethics Statement

All human samples were obtained with informed consent. Consent was also obtained from the participants to report individual patient data. The study protocols were approved by the Ethical Review Committee of Zhongshan Hospital affiliated to Fudan University. The patients/participants provided their written informed consent to participate in this study. The animal study was reviewed and approved by the Ethical Review Committee of Zhongshan Hospital affiliated to Fudan University. All animals received humane care according to the criteria outlined in the Guide for the Care and Use of Laboratory Animals prepared by the National Academy of Sciences and published by the National Institutes of Health. Written informed consent was obtained from the owners for the participation of their animals in this study.

## Author Contributions

SL and XY designed the study. CF, LJ, and SH performed the study. ZL and SH participated in parts of the experiments and analysis of the data. CF, SD, and WZ wrote the paper. All authors read and approved the final manuscript.

### Conflict of Interest

The authors declare that the research was conducted in the absence of any commercial or financial relationships that could be construed as a potential conflict of interest.

## Footnotes

^1^http://xena.ucsc.edu/welcome-to-ucsc-xena/
