# Supplementary material for: Activation of the IL-4/STAT6 Signaling Pathway Promotes Lung Cancer Progression by Increasing M2 Myeloid Cells
Source: Front Immunol. 2019 Nov 13;10:2638. doi: 10.3389/fimmu.2019.02638 (PMC6863933; doi:10.3389/fimmu.2019.02638)
Supplement: Supplementary file 1 [file Data_Sheet_1.pdf]

Supplementary Material

Supplementary Figures

Figure legends

Supplementary Figure S1. Effect of siRNA on the expression of STAT6

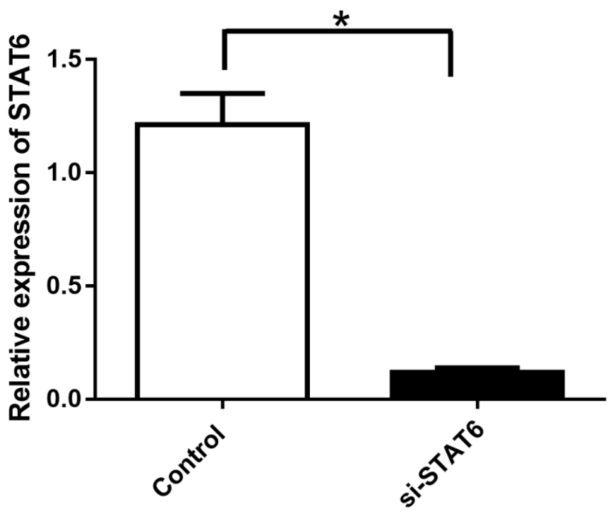

siRNA could efficiently reduce the mRNA expression of STAT6 by 97.5%. The knock down efficiencies of si-STAT6 in SPC-A1 was 97.5%, as verified by RT-PCR.

Supplementary Figure S2. The correlation between the expression of STAT6 and survival rate

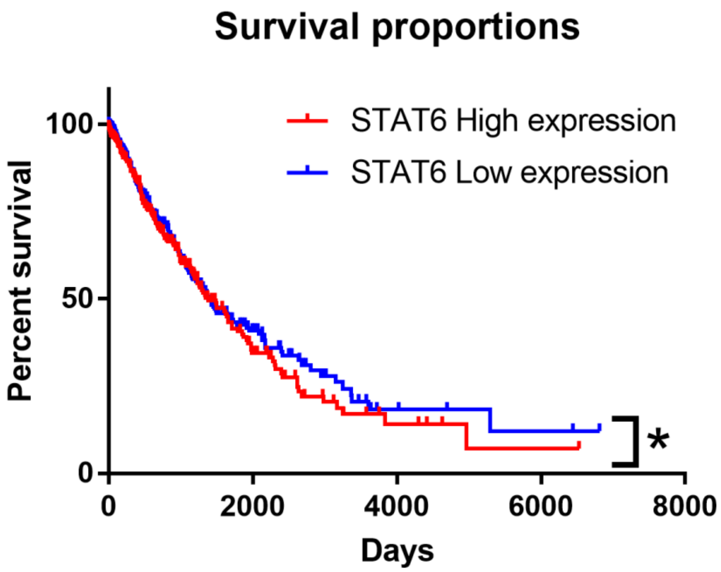

Data from the UCSC Cancer Genomics Browser[1]([1]http://xena.ucsc.edu/welcome-to-ucsc-xena/)indicate that A higher level of STAT6 expression in lung cancer is correlated with poorer survival.

[1] http://xena.ucsc.edu/welcome-to-ucsc-xena/

Supplementary Figure S3. TUNEL staining and Ki67 staining in tumors from STAT6$^{-/-}$ and WT mice

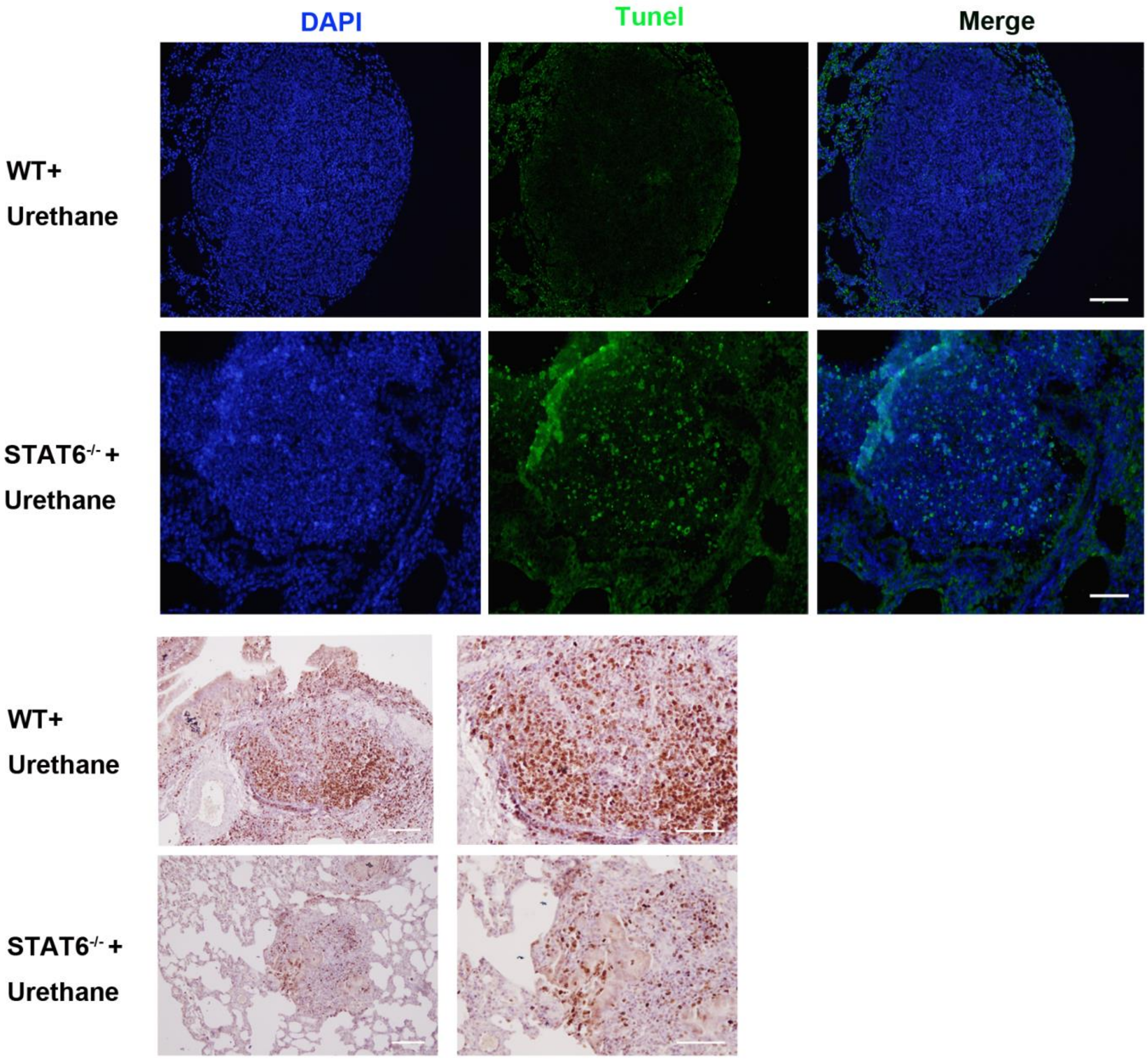

The rate of TUNEL staining (upper figures) measured by immunofluorescence in the group of WT and STAT6 tumor bearing mice. Positive immunohistochemical staining of Ki67 (lower figures) in tumors from WT and STAT6$^{-/-}$ mice. Tissue was obtained in six month after urethane incubation.

Supplementary Figure S4. Analysis of expression and function of CD4^{+} T cells in STAT6$^{-/-}$ tumor-bearing mice.

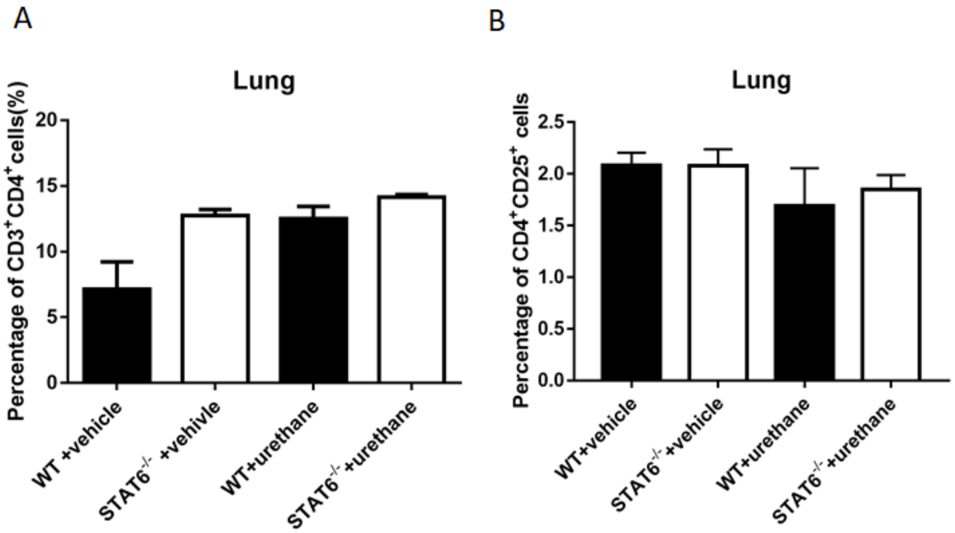

(A) Percentage of $CD3^{+}CD4^{+}$ cells in the lung of mice. **(B)** Percentage of $CD4^{+}$ $CD25^{+}$ cells in the lung of mice.

Supplementary Figure S5. Representative figure of pulmonary adenocarcinoma in the lung cancer patients.

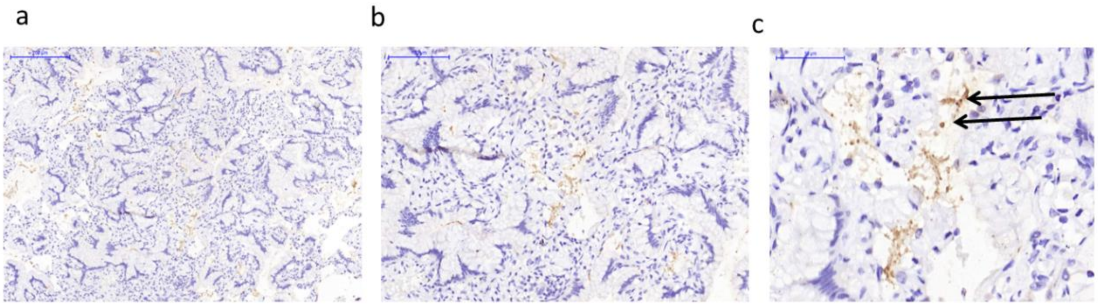

a. The bar measures 200 μ m, b. The bar measures 100 μ m. c. The bar measures 50 μ m. Black arrows pointed to the positive staining of STAT6.

Supplementary Figure S6. Percentage of Ly6G$^+$Ly6C$^-$ cells and CD11C$^+$ cells among CD11b$^+$ cells in the blood, bone marrow and the lung of mice.

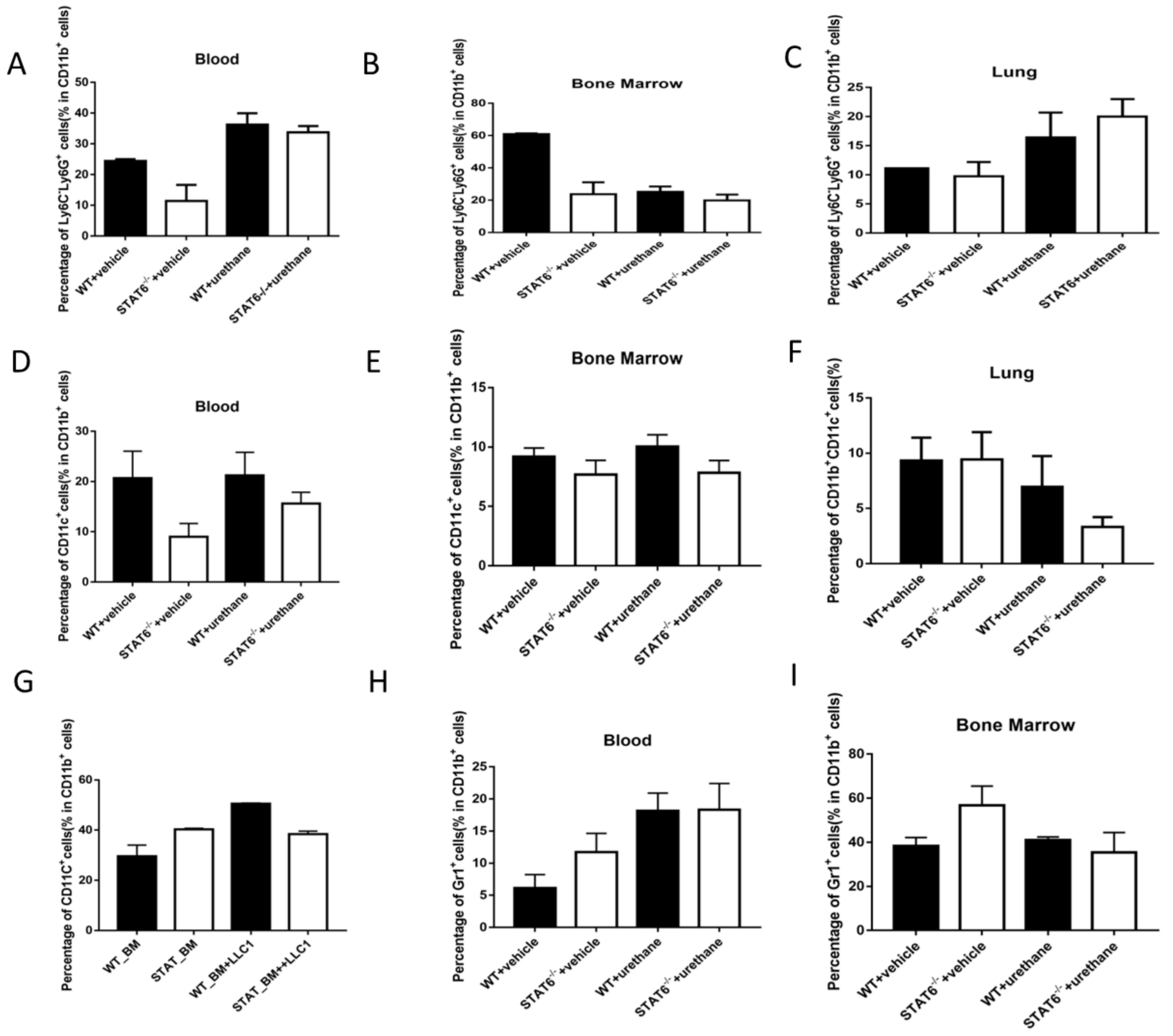

A-C: Percentage of Ly6C$^-$Ly6G$^+$ cells among CD11b$^+$ cells in the blood (A), bone marrow (B), and the lung (C). A-C: Percentage of CD11C$^+$ cells among CD11b$^+$ cells in the blood (D), bone marrow (E), and the lung (F). G: bone marrow cells co-cultured with LLC1 cells or same number of bone marrow cells using transwell system. Percentage of CD11C$^+$ cells among CD11b$^+$ cells were analyzed of the bone marrow cells in the lower side. H-I: Percentage of Gr1$^+$ cells among CD11b$^+$ cells in the blood (H), bone marrow (I).
